# 2-{(*E*)-[(3-Iodo-4-methyl­phen­yl)imino]­meth­yl}-4-(trifluoro­meth­oxy)phenol

**DOI:** 10.1107/S1600536812026876

**Published:** 2012-06-20

**Authors:** Merve Pekdemir, Şamil Işık, Ayşen Alaman Ağar

**Affiliations:** a Department of Physics, Faculty of Arts and Sciences, Ondokuz Mayıs University, Kurupelit, TR-55139 Samsun, Turkey; b Department of Chemistry, Faculty of Arts and Sciences, Ondokuz Mayıs University, Kurupelit, TR-55139 Samsun, Turkey

## Abstract

The title compound, C_15_H_11_F_3_INO_2_, adopts the enol–imine tautomeric form. The mol­ecule displays an *E* conformation with respect to the imine C=N double bond. The dihedral angle between the two benzene rings is 12.4 (2)°. The mol­ecular conformation is stabilized by an intra­molecular O—H⋯N hydrogen bond, which generates an *S*(6) ring motif. The trifluoro­meth­oxy­phenyl group is disordered over two sites with an occupancy ratio of 0.621 (6):0.379 (6). The crystal structure features C—H⋯π inter­actions.

## Related literature

For generic history to the use of Schiff bases and their biological activity, see: Tarafder *et al.* (2002 ▶); Cukurovali *et al.* (2002 ▶); Ali *et al.* (2002 ▶). Schiff base compounds can be classified by their photochromic and thermochromic characteristics, see: Alarcon *et al.* (1999 ▶); Cohen *et al.* (1964 ▶); Gül *et al.* (2007 ▶); Hadjoudis *et al.* (1987 ▶); Şahin *et al.* (2005 ▶); Xu *et al.* (1994 ▶). For related structures, see: Ağar *et al.* 2010 ▶); Ceylan *et al.* (2011 ▶); Demirtaş *et al.* (2009) ▶; Tecer *et al.* (2010 ▶). For hydrogen-bond motifs, see: Bernstein *et al.* (1995 ▶).
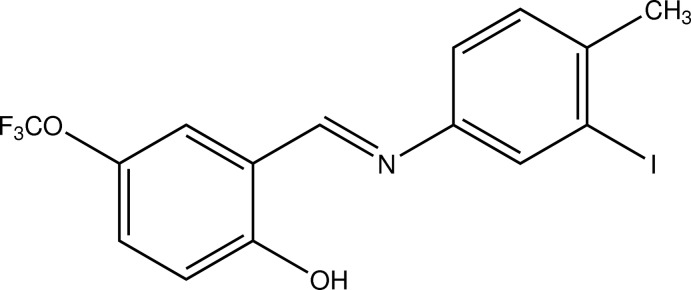


## Experimental

### 

#### Crystal data

C_15_H_11_F_3_INO_2_
*M* *_r_* = 421.15
Triclinic, 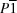

*a* = 4.6733 (3) Å
*b* = 6.6441 (5) Å
*c* = 25.2825 (19) Å
α = 86.970 (6)°
β = 86.386 (6)°
γ = 78.087 (5)°
*V* = 765.95 (10) Å^3^
*Z* = 2
Mo *K*α radiation
μ = 2.13 mm^−1^
*T* = 296 K
0.80 × 0.38 × 0.10 mm

#### Data collection

Stoe IPDS 2 diffractometer
Absorption correction: integration (*X-RED32*; Stoe & Cie, 2002 ▶) *T*_min_ = 0.389, *T*_max_ = 0.833
9671 measured reflections
3237 independent reflections
2806 reflections with *I* > 2σ(*I*)
*R*_int_ = 0.066

#### Refinement

*R*[*F* ^2^ > 2σ(*F* ^2^)] = 0.038
*wR*(*F* ^2^) = 0.107
*S* = 1.04
3237 reflections
198 parameters
38 restraints
H-atom parameters constrained
Δρ_max_ = 0.71 e Å^−3^
Δρ_min_ = −0.58 e Å^−3^

### 

Data collection: *X-AREA* (Stoe & Cie, 2002 ▶); cell refinement: *X-AREA*; data reduction: *X-RED32* (Stoe & Cie, 2002 ▶); program(s) used to solve structure: *SHELXS97* (Sheldrick, 2008 ▶); program(s) used to refine structure: *SHELXL97* (Sheldrick, 2008 ▶); molecular graphics: *ORTEP-3 for Windows* (Farrugia, 1997 ▶); software used to prepare material for publication: *WinGX* (Farrugia, 1999 ▶).

## Supplementary Material

Crystal structure: contains datablock(s) I, global. DOI: 10.1107/S1600536812026876/lr2067sup1.cif

Structure factors: contains datablock(s) I. DOI: 10.1107/S1600536812026876/lr2067Isup2.hkl

Supplementary material file. DOI: 10.1107/S1600536812026876/lr2067Isup3.cml

Additional supplementary materials:  crystallographic information; 3D view; checkCIF report

## Figures and Tables

**Table 1:** Hydrogen-bond geometry (Å, °) *Cg* is the centroid of the C1–C6 benzene ring.

*D*—H⋯*A*	*D*—H	H⋯*A*	*D*⋯*A*	*D*—H⋯*A*
O1—H1⋯N1	0.82	1.90	2.628 (4)	147
C15—H15*B*⋯*Cg* ^i^	0.96	2.85	3.570 (5)	133

Symmetry code: (i) 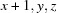.

**Fig. 1.**
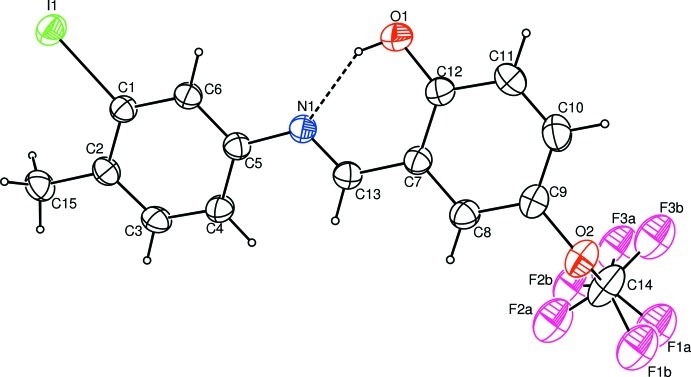
The molecular structure of the title compound. Displacement ellipsoids are drawn at the 30% probability.

## supplementary crystallographic information

### Comment

Schiff base complexes are of major interests for inorganic and bioinorganic chemistry. To the best of our knowledge, in recent years, Schiff base ligands have demonstrated important biological activities and new samples have been tested for their antitumor, antimicrobial and antiviral activities (Tarafder *et al.*, 2002; Cukurovali *et al.*, 2002; Ali *et al.*, 2002).

Schiff base compounds display interesting photochromic and thermochromic properties in the solid state and can be classified in terms of these features (Cohen *et al.*, 1964). Photo- and thermochromism arise *via* H-atom transfer from an hydroxy O atom to the imine N atom (Hadjoudis *et al.*, 1987; Xu *et al.*, 1994). Such proton- exchanging materials can be used for the design of various molecular electronic devices (Alarcon *et al.*, 1999). In general, Schiff bases display two possible tautomeric forms, the phenol-imine (OH) and the keto-amine (NH) forms. Depending on the tautomers, two sort of intramolecular hydrogen bonds are observed in Schiff bases: O—H···N in phenol-imine (Gül *et al.*, 2007) and N—H···O in keto-amine tautomers (Şahin *et al.*, 2005).

As an extension of the work on the structural characterization of Schiff base compounds, the crystal structure of the title compound is reported here. Our researchs show that compound (I) adopts the phenol-imine tautomeric form. The molecular structure of the title compound is shown in Fig.1. The molecule contains two aromatic rings linked through a imine group. The dihedral angle between the two benzene ring is 12.4 (2)°. The C5—N1—C13—C7 torsion angle is 179.4 (3)°. The C13═N1 bond distance [1.272 (5) Å] is consistent with related structures (Aǧar *et al.*, 2010; Tecer *et al.*, 2010; Ceylan *et al.*, 2011; Demirtaş *et al.*, 2009).

The trifluoromethyl group is disordered and have been refined as such (see refinement details). The F atoms are disordered over two positions with refined site occupancies of 0.621 (6): 0.379 (6).

Fig.1 additionally shows a strong intramolecular hyrogen bond (O1—H1···N1) can be defined as an S(6) motif (Bernstein *et al.*, 1995). The molecule are packaged by C—H···π interactions.

### Experimental

The title compound l was prepared by mixing a solution 2-hydroxy-5- (trifluoromethoxy)benzaldehyde (0.0107 g 0.052 mmol) in 20 ml ethanol with a solution of 3-iodo-4-methylaniline (0.0121 g 0.052 mmol) in 20 ml ethanol and refluxing the resulting mixture by 1 h under stirring. The crystals of 2-{(*E*)-[(3-iodo-4-methylphenyl)imino]methyl}-4-(trifluoromethoxy)phenol suitable for X-ray analysis were obtained from ethylalcohol by slow evaporation (yield %63; m.p 88–90 °C).

### Refinement

The H1 atom was located in a difference map and refined subject to a *DFIX* (*SHELXL97*; Sheldrick, 2008) restraint of O—H=0.82 (2) Å. All other H atoms were placed in calculated positions and constrained to ride on their parents atoms, with C—H = 0.93–0.96 Å and *U*_iso_(H) = 1.2*U*_eq_(C) or 1.5*U*_eq_(C).

### Crystal data

**Table:**

C_15_H_11_F_3_INO_2_	*Z* = 2
*M**_r_* = 421.15	*F*(000) = 408
Triclinic, *P*1	*D*_x_ = 1.826 Mg m^−^^3^
Hall symbol: -P 1	Mo *K*α radiation, λ = 0.71073 Å
*a* = 4.6733 (3) Å	Cell parameters from 18957 reflections
*b* = 6.6441 (5) Å	θ = 1.6–27.3°
*c* = 25.2825 (19) Å	µ = 2.13 mm^−^^1^
α = 86.970 (6)°	*T* = 296 K
β = 86.386 (6)°	PLATE, yellow
γ = 78.087 (5)°	0.80 × 0.38 × 0.10 mm
*V* = 765.95 (10) Å^3^	

### Data collection

**Table:**

Stoe IPDS 2 diffractometer	3237 independent reflections
Radiation source: fine-focus sealed tube	2806 reflections with *I* > 2σ(*I*)
Plane graphite monochromator	*R*_int_ = 0.066
Detector resolution: 6.67 pixels mm^-1^	θ_max_ = 26.8°, θ_min_ = 1.6°
rotation method scans	*h* = −5→5
Absorption correction: integration (*X-RED32*; Stoe & Cie, 2002)	*k* = −8→8
*T*_min_ = 0.389, *T*_max_ = 0.833	*l* = −31→31
9671 measured reflections	

### Refinement

**Table:**

Refinement on *F*^2^	Primary atom site location: structure-invariant direct methods
Least-squares matrix: full	Secondary atom site location: difference Fourier map
*R*[*F*^2^ > 2σ(*F*^2^)] = 0.038	Hydrogen site location: inferred from neighbouring sites
*wR*(*F*^2^) = 0.107	H-atom parameters constrained
*S* = 1.04	*w* = 1/[σ^2^(*F*_o_^2^) + (0.0721*P*)^2^ + 0.1093*P*] where *P* = (*F*_o_^2^ + 2*F*_c_^2^)/3
3237 reflections	(Δ/σ)_max_ = 0.002
198 parameters	Δρ_max_ = 0.71 e Å^−^^3^
38 restraints	Δρ_min_ = −0.58 e Å^−^^3^

### Special details

**Table:**

Geometry. All e.s.d.'s (except the e.s.d. in the dihedral angle between two l.s. planes) are estimated using the full covariance matrix. The cell e.s.d.'s are taken into account individually in the estimation of e.s.d.'s in distances, angles and torsion angles; correlations between e.s.d.'s in cell parameters are only used when they are defined by crystal symmetry. An approximate (isotropic) treatment of cell e.s.d.'s is used for estimating e.s.d.'s involving l.s. planes.
Refinement. Refinement of *F*^2^ against ALL reflections. The weighted *R*-factor *wR* and goodness of fit *S* are based on *F*^2^, conventional *R*-factors *R* are based on *F*, with *F* set to zero for negative *F*^2^. The threshold expression of *F*^2^ > σ(*F*^2^) is used only for calculating *R*-factors(gt) *etc*. and is not relevant to the choice of reflections for refinement. *R*-factors based on *F*^2^ are statistically about twice as large as those based on *F*, and *R*- factors based on ALL data will be even larger.

### Fractional atomic coordinates and isotropic or equivalent isotropic displacement parameters (Å^2^)

**Table:**

	*x*	*y*	*z*	*U*_iso_*/*U*_eq_	Occ. (<1)
C15	0.9104 (9)	0.8097 (7)	0.57659 (18)	0.0655 (10)	
H15A	0.9969	0.6685	0.5849	0.098*	
H15B	1.0566	0.8921	0.5770	0.098*	
H15C	0.8333	0.8211	0.5420	0.098*	
C14	−0.6844 (15)	0.7327 (12)	0.9450 (2)	0.0967 (18)	
I1	0.59478 (6)	1.29948 (4)	0.557019 (11)	0.06978 (14)	
O1	−0.3927 (8)	1.3987 (4)	0.77435 (14)	0.0774 (9)	
H1	−0.2581	1.3429	0.7544	0.116*	
N1	−0.0211 (6)	1.0922 (4)	0.73088 (12)	0.0511 (6)	
C1	0.5067 (7)	1.0843 (5)	0.61658 (13)	0.0487 (7)	
C5	0.2116 (7)	1.0139 (5)	0.69393 (13)	0.0491 (7)	
C6	0.2828 (7)	1.1499 (5)	0.65460 (14)	0.0498 (7)	
H6	0.1808	1.2858	0.6535	0.060*	
O2	−0.8277 (7)	0.8215 (5)	0.90303 (13)	0.0752 (8)	
C3	0.5939 (8)	0.7512 (5)	0.65756 (16)	0.0578 (8)	
H3	0.6984	0.6160	0.6593	0.069*	
C13	−0.1359 (8)	0.9712 (5)	0.76217 (14)	0.0528 (7)	
H13	−0.0655	0.8301	0.7601	0.063*	
C7	−0.3726 (7)	1.0436 (5)	0.80106 (14)	0.0501 (7)	
C4	0.3711 (8)	0.8124 (6)	0.69536 (15)	0.0566 (8)	
H4	0.3276	0.7190	0.7218	0.068*	
C12	−0.4914 (8)	1.2548 (6)	0.80557 (15)	0.0575 (8)	
C8	−0.4832 (8)	0.9026 (6)	0.83407 (16)	0.0563 (8)	
H8	−0.4079	0.7626	0.8309	0.068*	
C2	0.6688 (7)	0.8836 (5)	0.61677 (15)	0.0520 (7)	
C10	−0.8238 (10)	1.1762 (8)	0.87578 (19)	0.0725 (11)	
H10	−0.9750	1.2198	0.9009	0.087*	
C9	−0.7043 (8)	0.9682 (6)	0.87166 (15)	0.0602 (9)	
C11	−0.7204 (10)	1.3145 (7)	0.8433 (2)	0.0716 (11)	
H11	−0.8034	1.4536	0.8461	0.086*	
F1A	−0.830 (2)	0.6351 (14)	0.9751 (3)	0.1137 (11)	0.621 (6)
F2A	−0.4368 (18)	0.5983 (14)	0.9256 (3)	0.1137 (11)	0.621 (6)
F3A	−0.5481 (19)	0.8352 (13)	0.9714 (3)	0.1137 (11)	0.621 (6)
F1B	−0.818 (4)	0.571 (2)	0.9597 (5)	0.1137 (11)	0.379 (6)
F2B	−0.428 (3)	0.704 (2)	0.9445 (5)	0.1137 (11)	0.379 (6)
F3B	−0.758 (3)	0.8901 (18)	0.9804 (4)	0.1137 (11)	0.379 (6)

### Atomic displacement parameters (Å^2^)

**Table:**

	*U*^11^	*U*^22^	*U*^33^	*U*^12^	*U*^13^	*U*^23^
C15	0.057 (2)	0.064 (2)	0.072 (2)	−0.0052 (17)	0.0091 (18)	−0.0137 (19)
C14	0.102 (4)	0.128 (5)	0.074 (3)	−0.062 (4)	−0.007 (3)	0.026 (3)
I1	0.0703 (2)	0.0657 (2)	0.0686 (2)	−0.01119 (13)	0.01306 (13)	0.00966 (13)
O1	0.088 (2)	0.0503 (14)	0.083 (2)	0.0003 (13)	0.0198 (16)	0.0101 (14)
N1	0.0505 (14)	0.0500 (14)	0.0493 (14)	−0.0040 (12)	0.0000 (12)	0.0015 (12)
C1	0.0473 (16)	0.0482 (15)	0.0500 (16)	−0.0087 (13)	−0.0021 (13)	−0.0008 (13)
C5	0.0475 (16)	0.0483 (16)	0.0497 (17)	−0.0050 (13)	−0.0039 (13)	−0.0014 (13)
C6	0.0476 (16)	0.0457 (15)	0.0530 (17)	−0.0029 (13)	−0.0019 (13)	−0.0002 (13)
O2	0.0662 (16)	0.091 (2)	0.0705 (18)	−0.0272 (15)	−0.0008 (14)	0.0153 (16)
C3	0.0545 (19)	0.0446 (16)	0.068 (2)	0.0029 (14)	−0.0012 (16)	−0.0029 (15)
C13	0.0545 (18)	0.0483 (16)	0.0541 (18)	−0.0072 (14)	−0.0030 (14)	−0.0010 (14)
C7	0.0473 (16)	0.0506 (16)	0.0507 (17)	−0.0072 (13)	−0.0030 (13)	0.0020 (13)
C4	0.0569 (19)	0.0497 (17)	0.058 (2)	−0.0016 (15)	0.0014 (15)	0.0063 (15)
C12	0.061 (2)	0.0515 (17)	0.0570 (19)	−0.0071 (15)	0.0012 (16)	0.0032 (15)
C8	0.0537 (18)	0.0540 (18)	0.060 (2)	−0.0086 (15)	−0.0038 (15)	0.0040 (15)
C2	0.0444 (16)	0.0538 (17)	0.0559 (18)	−0.0045 (14)	−0.0021 (14)	−0.0085 (15)
C10	0.064 (2)	0.082 (3)	0.067 (2)	−0.008 (2)	0.0115 (19)	−0.009 (2)
C9	0.0553 (19)	0.072 (2)	0.0546 (19)	−0.0175 (17)	−0.0003 (15)	0.0035 (17)
C11	0.068 (2)	0.061 (2)	0.079 (3)	0.0028 (18)	0.010 (2)	−0.009 (2)
F1A	0.1195 (19)	0.136 (3)	0.090 (2)	−0.043 (2)	−0.010 (2)	0.0223 (18)
F2A	0.1195 (19)	0.136 (3)	0.090 (2)	−0.043 (2)	−0.010 (2)	0.0223 (18)
F3A	0.1195 (19)	0.136 (3)	0.090 (2)	−0.043 (2)	−0.010 (2)	0.0223 (18)
F1B	0.1195 (19)	0.136 (3)	0.090 (2)	−0.043 (2)	−0.010 (2)	0.0223 (18)
F2B	0.1195 (19)	0.136 (3)	0.090 (2)	−0.043 (2)	−0.010 (2)	0.0223 (18)
F3B	0.1195 (19)	0.136 (3)	0.090 (2)	−0.043 (2)	−0.010 (2)	0.0223 (18)

### Geometric parameters (Å, º)

**Table:**

C15—C2	1.492 (5)	C5—C4	1.391 (5)
C15—H15A	0.9600	C6—H6	0.9300
C15—H15B	0.9600	O2—C9	1.415 (5)
C15—H15C	0.9600	C3—C4	1.380 (5)
C14—F2B	1.174 (16)	C3—C2	1.395 (6)
C14—F1A	1.236 (10)	C3—H3	0.9300
C14—F3A	1.263 (10)	C13—C7	1.454 (5)
C14—O2	1.336 (7)	C13—H13	0.9300
C14—F1B	1.371 (16)	C7—C8	1.380 (5)
C14—F3B	1.387 (12)	C7—C12	1.406 (5)
C14—F2A	1.387 (10)	C4—H4	0.9300
I1—C1	2.102 (3)	C12—C11	1.396 (6)
O1—C12	1.344 (5)	C8—C9	1.377 (6)
O1—H1	0.8200	C8—H8	0.9300
N1—C13	1.272 (5)	C10—C11	1.343 (7)
N1—C5	1.420 (4)	C10—C9	1.386 (6)
C1—C2	1.391 (5)	C10—H10	0.9300
C1—C6	1.392 (5)	C11—H11	0.9300
C5—C6	1.379 (5)		
			
C2—C15—H15A	109.5	C5—C6—C1	120.2 (3)
C2—C15—H15B	109.5	C5—C6—H6	119.9
H15A—C15—H15B	109.5	C1—C6—H6	119.9
C2—C15—H15C	109.5	C14—O2—C9	117.8 (4)
H15A—C15—H15C	109.5	C4—C3—C2	122.7 (3)
H15B—C15—H15C	109.5	C4—C3—H3	118.6
F2B—C14—F1A	122.8 (10)	C2—C3—H3	118.6
F2B—C14—F3A	56.8 (8)	N1—C13—C7	122.9 (3)
F1A—C14—F3A	110.3 (7)	N1—C13—H13	118.6
F2B—C14—O2	120.6 (8)	C7—C13—H13	118.6
F1A—C14—O2	113.4 (6)	C8—C7—C12	119.4 (3)
F3A—C14—O2	119.7 (6)	C8—C7—C13	119.4 (3)
F2B—C14—F1B	118.3 (11)	C12—C7—C13	121.2 (3)
F1A—C14—F1B	25.2 (7)	C3—C4—C5	120.0 (3)
F3A—C14—F1B	131.3 (8)	C3—C4—H4	120.0
O2—C14—F1B	103.9 (7)	C5—C4—H4	120.0
F2B—C14—F3B	100.5 (10)	O1—C12—C11	119.7 (4)
F1A—C14—F3B	86.9 (7)	O1—C12—C7	121.9 (3)
F3A—C14—F3B	43.6 (6)	C11—C12—C7	118.4 (4)
O2—C14—F3B	100.3 (7)	C9—C8—C7	120.3 (4)
F1B—C14—F3B	111.8 (9)	C9—C8—H8	119.8
F2B—C14—F2A	39.0 (8)	C7—C8—H8	119.8
F1A—C14—F2A	108.9 (8)	C1—C2—C3	115.9 (3)
F3A—C14—F2A	95.7 (7)	C1—C2—C15	123.3 (4)
O2—C14—F2A	106.9 (6)	C3—C2—C15	120.7 (3)
F1B—C14—F2A	90.9 (9)	C11—C10—C9	119.7 (4)
F3B—C14—F2A	139.0 (8)	C11—C10—H10	120.1
C12—O1—H1	109.5	C9—C10—H10	120.1
C13—N1—C5	120.7 (3)	C8—C9—C10	120.4 (4)
C2—C1—C6	122.3 (3)	C8—C9—O2	119.7 (4)
C2—C1—I1	119.9 (3)	C10—C9—O2	119.7 (4)
C6—C1—I1	117.8 (2)	C10—C11—C12	121.7 (4)
C6—C5—C4	118.8 (3)	C10—C11—H11	119.1
C6—C5—N1	116.8 (3)	C12—C11—H11	119.1
C4—C5—N1	124.4 (3)		
			
C13—N1—C5—C6	167.1 (3)	C8—C7—C12—C11	−0.5 (6)
C13—N1—C5—C4	−13.6 (5)	C13—C7—C12—C11	179.3 (4)
C4—C5—C6—C1	1.2 (5)	C12—C7—C8—C9	−1.1 (6)
N1—C5—C6—C1	−179.4 (3)	C13—C7—C8—C9	179.1 (3)
C2—C1—C6—C5	−0.9 (5)	C6—C1—C2—C3	−0.1 (5)
I1—C1—C6—C5	178.6 (2)	I1—C1—C2—C3	−179.6 (3)
F2B—C14—O2—C9	−31.7 (14)	C6—C1—C2—C15	−179.8 (3)
F1A—C14—O2—C9	168.1 (7)	I1—C1—C2—C15	0.7 (5)
F3A—C14—O2—C9	35.1 (10)	C4—C3—C2—C1	0.7 (6)
F1B—C14—O2—C9	−167.2 (8)	C4—C3—C2—C15	−179.6 (4)
F3B—C14—O2—C9	77.1 (7)	C7—C8—C9—C10	1.8 (6)
F2A—C14—O2—C9	−71.9 (7)	C7—C8—C9—O2	176.2 (3)
C5—N1—C13—C7	179.4 (3)	C11—C10—C9—C8	−0.9 (7)
N1—C13—C7—C8	−179.2 (3)	C11—C10—C9—O2	−175.2 (4)
N1—C13—C7—C12	1.0 (5)	C14—O2—C9—C8	81.3 (6)
C2—C3—C4—C5	−0.3 (6)	C14—O2—C9—C10	−104.3 (6)
C6—C5—C4—C3	−0.7 (5)	C9—C10—C11—C12	−0.7 (8)
N1—C5—C4—C3	−180.0 (3)	O1—C12—C11—C10	−179.3 (4)
C8—C7—C12—O1	−179.8 (4)	C7—C12—C11—C10	1.4 (7)
C13—C7—C12—O1	0.0 (6)		

### Hydrogen-bond geometry (Å, º)

Cg is the centroid of the C1–C6 benzene ring.

**Table:**

*D*—H···*A*	*D*—H	H···*A*	*D*···*A*	*D*—H···*A*
O1—H1···N1	0.82	1.90	2.628 (4)	147
C15—H15*B*···*Cg*^i^	0.96	2.85	3.570 (5)	133

Symmetry code: (i) *x*+1, *y*, *z*.
